# Genome-wide analysis of a cellular exercise model based on electrical pulse stimulation

**DOI:** 10.1038/s41598-022-25758-2

**Published:** 2022-12-08

**Authors:** Bora Lee, Seon Kyu Kim, Yeo Jin Shin, Young Hoon Son, Jae Won Yang, Seung-Min Lee, Yong Ryul Yang, Kwang-Pyo Lee, Ki-Sun Kwon

**Affiliations:** 1 Aging Convergence Research Center, Korea Research Institute of Bioscience and Biotechnology (KRIBB), Daejeon, 34141 Republic of Korea; 2 Personalized Genomic Medicine Research Center, Korea Research Institute of Bioscience and Biotechnology (KRIBB), Daejeon, 34141 Republic of Korea; 3 Department of Bioinformatics, KRIBB School of Bioscience, Korea University of Science and Technology (UST), Daejeon, 34113 Republic of Korea; 4 Department of Biomolecular Science, KRIBB School of Bioscience, Korea University of Science and Technology (UST), Daejeon, 34113 Republic of Korea; 5 Department of Functional Genomics, KRIBB School of Bioscience, Korea University of Science and Technology (UST), Daejeon, 34113 Republic of Korea; 6 Aventi Inc., Daejeon, 34141 Republic of Korea

**Keywords:** Cell biology, Molecular biology

## Abstract

Skeletal muscle communicates with other organs via myokines, which are secreted by muscle during exercise and exert various effects. Despite much investigation of the exercise, the underlying molecular mechanisms are still not fully understood. Here, we applied an in vitro exercise model in which cultured C2C12 myotubes were subjected to electrical pulse stimulation (EPS), which mimics contracting muscle. Based on the significantly up- and down-regulated genes in EPS, we constructed an in silico model to predict exercise responses at the transcriptional level. The in silico model revealed similarities in the transcriptomes of the EPS and exercised animals. Comparative analysis of the EPS data and exercised mouse muscle identified putative biomarkers in exercise signaling pathways and enabled to discover novel exercise-induced myokines. Biochemical analysis of selected exercise signature genes in muscle from exercised mice showed that EPS mimics in vivo exercise, at least in part, at the transcriptional level. Consequently, we provide a novel myokine, Amphiregulin (AREG), up-regulated both in vitro and in vivo, that would be a potential target for exercise mimetics.

## Introduction

Exercise is essential for a healthy life. An appropriate amount of exercise delays the onset of chronic diseases such as type 2 diabetes^1^, cardiovascular disease^2^, Alzheimer’s disease^3^, and aging in a non-pharmacological manner. Exercise training reduces the risk of cancer by inhibiting cancer cell proliferation^4,5^ and reduces the risk of infection^6–8^.

Exercise exerts multiple physiological, metabolic, and immunological effects, and skeletal muscle can communicate with other organs such as the brain, heart, liver, and adipose tissue during exercise. This inter-organ communication is mediated by autocrine, paracrine, or endocrine crosstalk via myokines produced by skeletal muscle^9,10^. Transcriptomics and proteomics enable discovery of novel myokines using in vivo and in vitro systems. For example, Irisin was discovered using Affymetrix-based gene expression arrays in peroxisome proliferator-activated receptor gamma coactivator 1-alpha (PPARGC1A)-overexpressing mouse muscle^11^, and was validated in human by mass spectrometry^12^. Numerous candidate myokines have been identified, but it is unclear whether changes in their plasma levels are driven by increased expression in skeletal muscle or other organs.

Electrical pulse stimulation (EPS) of cultured myotubes mimics muscle contraction in vivo. Applying EPS to differentiated myotubes mimics motor neuron activation in muscle fibers and induces myotube contraction in vitro^13^. EPS also has the metabolic properties of in vivo exercise, such as increased ATP production via glycolysis and lipolysis as well as branched chain amino acid (BCAA) catabolism^14–16^. Furthermore, EPS has been used to identify novel myokines and to determine whether they are de novo synthesized in muscle cells and secreted by muscle cells. This approach eliminates the paracrine or endocrine effect of other organs, allowing assessment of the intrinsic or autocrine effect of muscle contraction. Using EPS, CXC motif chemokine ligand 1 (CXCL1)^17^, CXC motif chemokine ligand 5 (CXCL5)^18^, and ATP synthase inhibitory factor 1 (IF1)^19^ were identified as exercise-induced myokines in C2C12 myotubes. In human skeletal muscle cells, pigment epithelium derived factor (PEDF)^20^, leukemia inhibitory factor (LIF)^21^, chitinase-3-like protein 1 (CHI3L1)^22^, thymosin β4 (TMSB4X)^23^, and R-spondin 3 (RSPO3)^24^ have also been identified as myokines. However, the degree to which EPS mimics in vivo exercise at the molecular level is unclear.

In this study, we developed an in vitro cell system to discover novel myokines and exercise biomarkers. We constructed in silico model using a deep-learning method, and examined whether the model can predict the effect of exercise in vivo. We selected putative exercise signature genes consistently expressed in vitro and in vivo and analyzed their expression in mouse muscle after exercise. The in silico model confirmed that the EPS cell system mimics in vivo exercise. Therefore, the EPS cell system could be used to identify exercise signaling mechanisms and candidate myokines, thereby facilitating the development of exercise mimetics.

## Results

### The EPS-based in silico model can predict the effect of in vivo exercise

**Figure 1:**
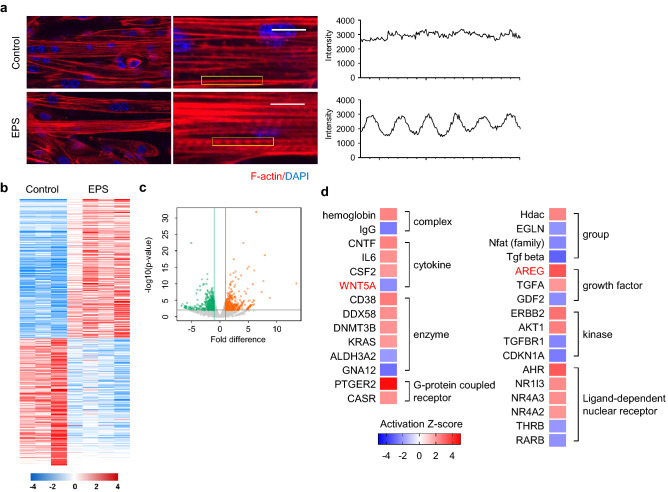
Effect of EPS in C2C12 myotubes. (**a**) (*Left*) Confocal images of C2C12 myotubes after applying EPS for 24 h, stained for F-actin (red) and DAPI (blue). Yellow bracket, portion used for intensity measurement (*right*). Scale bars, 10 μm. (**b**,**c**) DEGs between the control (n = 3) and EPS (n = 4) groups and volcano plot of DEGs. A total of 774 DEGs was identified (*P* < 0.01, fold change > 2). (**d**) Predicted upstream regulators from IPA with activation Z-scores ≥ 2 or ≤  − 2 and *P* < 0.05.

To identify exercise-induced signaling in muscle without interference by other organs, we used an in vitro contraction system that mimics muscle contraction by applying EPS for 24 h (1 ms pulse stimulus of 2 ms duration at 11.5 V and 1 Hz) in C2C12 myotubes. Because EPS reportedly enhances de novo sarcomere assembly^17,25^, we evaluated sarcomere structure development by staining actin filaments (F-actin) after EPS. Compared to the control, EPS resulted in a more prominent banded pattern of F-actin that ran longitudinally along myotubes (Fig. 1a). RNA sequencing (RNA-seq) showed that between the control and EPS groups, there were 774 differentially expressed genes (DEGs) (*P* < 0.01, fold change > 2), among which 406 were significantly up-regulated and 368 were significantly down-regulated (Fig. 1b,c, and Supplementary Table 1). To identify transcriptional regulators associated with exercise, we performed an ingenuity pathway analysis (IPA) of the 774 DEGs (Fig. 1d, Supplementary Fig. 1, and Supplementary Table 2). Among the up-regulated genes in the EPS group, forkhead box M1 (FOXM1; z-score = 3.503), amphiregulin (AREG; z-score = 3.337), and Wnt family member 5A (WNT5A; z-score = − 2.243) were identified as upstream regulators.

Although the exercise-induced factors interleukin-6 (IL-6) and PPARGC1A were not significantly induced in the EPS group compared to the unstimulated control group, they were classified as significantly activated “virtual transcriptional regulators” in the EPS group (IL-6 z-score = 2.151, PPARGC1A z-score = 2.789).

**Figure 2:**
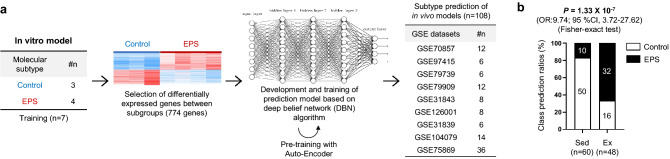
Development of a prediction model using a DBN algorithm. (**a**) Schematic of the molecular subtype in vitro exercise prediction model. Nine publicly available datasets (n = 108) were used to determine the predictive value of the model based on the H2O deep-learning method. (**b**) Prediction results obtained using the molecular subtype prediction model. Among 60 sedentary samples, 50 were predicted accurately as control samples. Among 48 exercise samples, 32 were predicted correctly as EPS samples.

We determined whether the EPS mimic in vivo exercise through transcriptome profiling. A deep-belief network (DBN) was used to predict exercise responses at the transcriptional level using RNA-seq data (Fig. 2a). In total, 774 DEGs in the RNA-seq data were pooled to form a classifier. The classifier was used to estimate the probability of an in vivo sample (sedentary [sed] or exercised [ex]) belonging to the control and EPS groups. To prevent overfitting of the classifier, the prediction model was pre-trained using an auto-encoder algorithm^26^. This model was applied to the publicly available in vivo datasets (Table 1). Despite the variability of the datasets, the classifier identified significant differences between the sed and ex groups; 83% of the sed samples belonged to the control group and 67% of the ex samples belonged to the EPS group (Fisher’s exact test, *p* = 1.33 × 10^−7^; Fig. 2b). Therefore, the EPS cell system mimics in vivo exercise at the transcriptional level.

**Table 1:** Publicly available in vivo dataset.

GEO accession	Type of experiment	Characteristics of samples	Exercise protocol	Type of muscle
GSE70857	Expression profiling by array	3 Months wild type (WT) (C57/BL6) male mice	Running wheel cage during 10 weeks	Not mentioned
GSE31839	Expression profiling by high-throughput sequencing	6 Months WT (C57BL/6) male mice	Running wheel cage during 12 weeks	Triceps brachii
GSE31843	Expression profiling by high-throughput sequencing	6 Months WT (C57BL/6) male mice	Running wheel cage during 12 weeks	Quadriceps femoris, Gastrocnemius/plantaris, Tibialis anterior, Triceps brachii
GSE79739	Expression profiling by high-throughput sequencing	4 Months WT (C57BL/6) male mice	3 Bouts of treadmill running, with each bout lasting for 1 h at 10 m/min and 1 h rest interval in between runs	Quadriceps femoris
GSE79907	Expression profiling by array	5 Weeks WT (SJ6/C57BL6) female mice	Single bout of downhill running (− 20°, 17 m/min, 40 min) on a treadmill	Gastrocnemius/Soleus complex
GSE97415	Expression profiling by array	12 Weeks WT (B6/129) male mice	Treadmill exercise consisting of a warmup [5 (10 m/min) and 5 min (15 m/min)] followed by 50 min (18 m/min) at a 5% incline	Plantaris
GSE126001	Expression profiling by high-throughput sequencing	20 Weeks WT (C57BL/6J) male mice	Endurance exercise trained for 5 weeks by daily treadmill running	Gastrocnemius
GSE104079	Expression profiling by array	6 Months WT (C57BL/6) male mice	Daily treadmill running exercise during 4 weeks	Soleus
GSE75869	Expression profiling by array	3 Months sex-matched (C57BL/6) mice	Treadmill exercise 3 times per week at 15 m/min for 45 min for 6 months	Quadriceps femoris

### In vitro exercise mimics in vivo exercise

Because the EPS cell system could recapitulate in vivo exercise at the transcriptional level, we next identified individual genes with common expression patterns in EPS and in vivo exercise.

**Figure 3:**
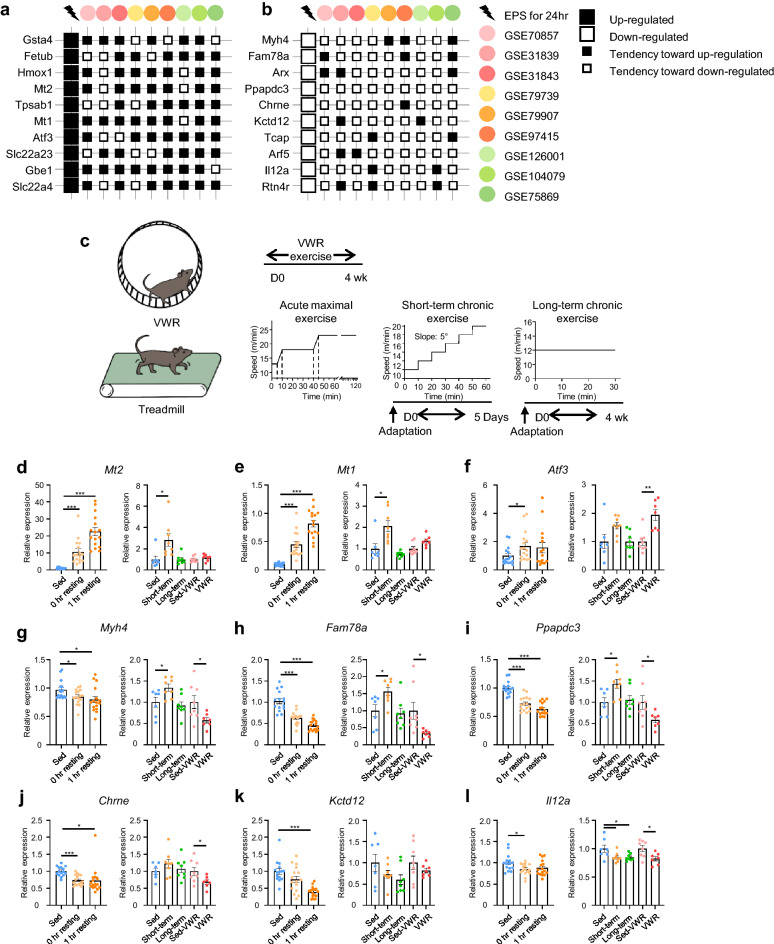
Expression of DEGs in exercised muscles. (**a**,**b**) Top 10 up- and down-regulated genes in the EPS group in the publicly available in vivo exercise datasets. Genes with log2-transformed read counts per million mapped fragments (CPM) values < 1 were excluded to enhance accuracy, and the genes were sorted according to their fold differences in expression between the two groups (control and EPS). (**c**) Exercise protocols for subsequent in vivo exercise. VWR was performed for 4 weeks ad libitum on running wheels. The acute maximal exercise was performed once until the mice were exhausted. The short-term chronic exercise was performed at a gradually increased speed for 5 days. The long-term chronic exercise was performed at the same speed for 4 weeks. (**d**–**l**) *Mt2*, *Mt1*, *Aft3*, *Myh4*, *Fam78a*, *Ppapdc3*, *Chrne*, *Kctd12*, and *Il12a* expression following the indicated types of exercise. For acute maximal exercise, sed (n = 15), 0 h resting (n = 16), and 1 h resting (n = 16) tibialis anterior (TA) muscles according to the resting time after exercise were analyzed. For chronic exercise, sed (n = 7), short-term (n = 8), and long-term (n = 8) gastrocnemius (GA) muscles were analyzed. For VWR exercise, sed-VWR (n = 8) and VWR (n = 7) GA muscles were analyzed. Means ± SEM; *P < 0.05, **P < 0.01, ***P < 0.001.

We performed a comparative analysis of the EPS data and publicly available datasets of exercised mouse muscle. Genes showing common expression patterns in at least six datasets were analyzed (Supplementary Table 3). The top 10 up- or down-regulated genes were verified by quantitative reverse transcription (qRT)-PCR in EPS samples except aristaless related homeobox (*Arx*) and reticulon 4 receptor (*Rtn4r*), which showed low abundance (Fig. 3a,b, and Supplementary Fig. 2). These genes are hereafter referred to as the exercise signature.

To determine whether the selected exercise signature genes (Fig. 3a) showed consistent up- or down-regulation in vivo, we evaluated their expression in mice performing voluntary wheel running (VWR) exercise, acute maximal exercise, and short- and long-term chronic exercise (Fig. 3c). Although the exercise protocol and muscle types were different from the in vivo exercise datasets, 3 of 10 up-regulated genes and 8 of 10 down-regulated genes showed similar expression patterns in the exercise models, particularly that for acute maximal exercise (Fig. 3d–i and Supplementary Fig. 3). Among the up-regulated genes, metallothionein 2A (*Mt2*) and *Mt1* were significantly up-regulated by acute maximal and short-term chronic exercise (Fig. 3d and e). In addition, activating transcription factor 3 (*Atf3*) was up-regulated by acute maximal and VWR exercise (Fig. 3f). Among the top 10 down-regulated genes, myosin heavy chain 4 (*Myh4*), family with sequence similarity 78 member A (*Fam78a*), phosphatidic acid phosphatase type 2 domain containing 3 (*Ppapdc3*), cholinergic receptor nicotinic epsilon subunit (*Chrne*), and potassium channel tetramerization domain containing 12 (*Kctd12*) expression gradually decreased after acute maximal exercise (Fig. 3g–k), while *Myh4*, *Fam78a*, *Ppapdc3*, and *Chrne* expression decreased after VWR exercise (Fig. 3g–j). Interleukin 12A (*Il12a*) was down-regulated in all exercise settings (Fig. 3l). Therefore, the EPS cell system reflects, at least in part, in vivo exercise.

### Identification of a putative myokine

**Figure 4:**
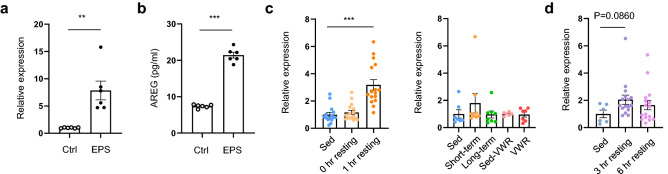
Areg, a candidate myokine biomarker, is up-regulated in EPS and acute maximally exercised muscles. (**a**) *Areg* expression in C2C12 myotubes after applying EPS for 24 h (n = 6). (**b**) Areg level in medium from C2C12 myotubes after applying EPS for 24 h (n = 6). (**c**) *Areg* expression in exercised muscles. For acute maximal exercise, sed (n = 15), 0 h resting (n = 16), and 1 h resting (n = 16) TA muscles according to the resting time after exercise were analyzed. For chronic exercise, sed (n = 7), short-term (n = 8), and long-term (n = 8) GA muscles were analyzed. For VWR exercise, sed-VWR (n = 6) and VWR (n = 7) GA muscles were analyzed. (**d**) *Areg* expression 3 and 6 h after acute maximal exercise. TA muscles from Sed (n = 6), 3 h resting (n = 16), 6 h resting (n = 16) mice were analyzed. Means ± SEM; **P < 0.01, ***P < 0.001.

EPS has been used to identify cytokines and myokines secreted by C2C12 cells^17–19,27^; therefore, we explored novel myokines up-regulated in an EPS cell system. To identify putative myokine candidates, we screened 774 DEGs in RNA-seq dataset for genes likely encoding secreted proteins annotated with the Gene Ontology (GO) term extracellular region (GO: 0005576) by GO analysis. Among the genes encoding secreted proteins, significantly up-regulated genes in response to EPS are listed in Table 2 (logCPM > − 1). In a literature search, we selected genes that are reportedly up-regulated during exercise or beneficial to muscle health. Among them, we focused on *Areg*, which was reported to be up-regulated in various exercise conditions in mice and humans and to enhance muscle repair (see discussion). *Areg* was highly up-regulated in the RNA-seq (Supplementary Table 1) and also predicted by IPA to be an activated transcriptional regulator (z-score = 3.337) (Fig. 1d). Expression of *Areg* was verified to be increased 7.7-fold by qRT-PCR in EPS myotubes (Fig. 4a). In mouse muscle, expression of *Areg* was increased 3.1-fold at 1 h after acute exercise and remained twofold increased after 3 h (Fig. 4c,d). To determine whether EPS triggers Areg secretion by C2C12 myotubes, we performed enzyme-linked immunosorbent assay (ELISA) on EPS conditioned medium; the Areg protein level was 2.9-fold increased (Fig. 4b). Considering that *Areg* mRNA is upregulated in exercised muscles as well as EPS-treated myotubes and that extracellular (secretion) Areg protein levels increased in EPS conditioned medium, it may be a novel myokine.

**Table 2:** EPS up-regulated genes annotated with the GO term extracellular region.

Gene name	Description	logFC
Prl2c2	Prolactin family 2, subfamily c, member 2	8.80
Prl2c3	Prolactin family 2, subfamily c, member 3	7.32
F13a1	Coagulation factor XIII, A1 subunit	3.34
Areg	Amphiregulin	2.99
Enpp2	Ectonucleotide pyrophosphatase/phosphodiesterase 2	2.77
Fetub	Fetuin beta	2.76
Ces2c	Carboxylesterase 2C	2.63
Nell2	NEL-like 2	2.62
Fgf1	Fibroblast growth factor 1	2.62
Nppb	Natriuretic peptide type B	2.57
Tpsab1	Tryptase alpha/beta 1	2.57
Angptl7	Angiopoietin-like 7	2.56
Prl2c5	Prolactin family 2, subfamily c, member 5	2.53
Gstm1	Glutathione S-transferase, mu 1	2.38
Gria1	Glutamate receptor, ionotropic, AMPA1 (alpha 1)	2.32
Cxcl5	Chemokine (C-X-C motif) ligand 5	2.32
Krt13	Keratin 13	2.22
Dkk2	Dickkopf WNT signaling pathway inhibitor 2	2.18
Il1b	Interleukin 1 beta	2.18
Procr	Protein C receptor, endothelial	2.12
Cxcl1	Chemokine (C-X-C motif) ligand 1	2.12
Gbp3	Guanylate binding protein 3	2.10
Apob	Apolipoprotein B	2.08
Glipr1	GLI pathogenesis-related 1 (glioma)	2.03
Gdf15	Growth differentiation factor 15	1.94
Aqp1	Aquaporin 1	1.93
Tacstd2	Tumor-associated calcium signal transducer 2	1.91
Sema4f.	Sema domain, immunoglobulin domain (Ig), TM domain, and short cytoplasmic domain	1.79
Serpinb9b	Serine (or cysteine) peptidase inhibitor, clade B, member 9b	1.79
Chit1	Chitinase 1 (chitotriosidase)	1.78
Fgfbp1	Fibroblast growth factor binding protein 1	1.65
Fth1	Ferritin heavy polypeptide 1	1.62
Xdh	Xanthine dehydrogenase	1.60
Serpinb1b	Serine (or cysteine) peptidase inhibitor, clade B, member 1b	1.52
Nid1	Nidogen 1	1.50
Apln	Apelin	1.44
Phospho1	Phosphatase, orphan 1	1.35
Kif23	Kinesin family member 23	1.27
Spint1	Serine protease inhibitor, Kunitz type 1	1.27
Gprc5a	G protein-coupled receptor, family C, group 5, member A	1.26
Vldlr	Very low density lipoprotein receptor	1.23
Serpinb1c	Serine (or cysteine) peptidase inhibitor, clade B, member 1c	1.23
Fam83d	Family with sequence similarity 83, member D	1.23
Tsku	Tsukushi, small leucine rich proteoglycan	1.22
Pkhd1	Polycystic kidney and hepatic disease 1	1.22
Lamc2	Laminin, gamma 2	1.16
Nmi	N-myc (and STAT) interactor	1.13
Wnt4	Wingless-type MMTV integration site family, member 4	1.12
Cd1d1	CD1d1 antigen	1.10
Sod1	Superoxide dismutase 1, soluble	1.09
Ftl1	Ferritin light polypeptide 1	1.09
Kif20a	Kinesin family member 20A	1.06
Cep55	Centrosomal protein 55	1.05
Olfm1	Olfactomedin 1	1.04
Hsph1	Heat shock 105 kDa/110 kDa protein 1	1.03
Cat	Catalase	1.03
Gdnf	Glial cell line derived neurotrophic factor	1.02
Serpinb9d	Serine (Or cysteine) peptidase inhibitor, clade B, member 9D	1.01
Wnt10b	Wingless-type MMTV integration site family, member 10B	1.01
Serpinb9c	Serine (or cysteine) peptidase inhibitor, clade B, member 9c	1.01

## Discussion

We established an in silico exercise model based on a DBN to predict exercise responses at the transcriptional level. EPS is used for in vitro validation of the effects of exercise because it enables modulation of the pulse frequency, voltage, and duration. EPS improves insulin sensitivity^28^, up-regulates slow-twitch related genes and myogenic transcription factors^29^, and increases the number of mitochondria^30^. However, the degree to which EPS mimics in vivo exercise at the molecular level is unclear. Using an in silico prediction model, we confirmed that EPS (1 ms pulse stimulus at 11.5 V and 1 Hz for 24 h) mimics in vivo exercise at the transcriptional level. We found novel exercise signature genes that could serve as candidate biomarkers in exercise signaling pathways and identified putative myokines.

The top 10 up- and down-regulated genes shared by the EPS and in vivo datasets were validated by analyzing mouse muscle post-exercise. The top 10 down-regulated genes (except low-abundance genes) were perfectly matched between the EPS and in vivo datasets in the context of acute maximal exercise, while none were consistent between the datasets in the context of in long-term chronic exercise, except *Il12a*. The long-term chronic exercise protocol may have been insufficient to elicit transcriptional changes. *Myh4*, which was down-regulated by VWR exercise and acute maximal exercise, is reportedly down-regulated in humans during aerobic and resistance exercise training^31^. *Ppapdc3/Plpp7* (inactive phospholipid phosphatase 7) is a muscle-specific regulator of nuclear envelope structure enriched in fast-twitch muscles^32^ and is associated with Emery-Dreifuss muscular dystrophy 3 (EDMD3)^33^. In this study, *Ppapdc3* was down-regulated consistently by EPS, acute maximal exercise, and VWR exercise. Because the genes down-regulated in EPS (except *Myh4*) have not been reported to be associated with exercise, further studies are needed to address the mechanisms and physiological roles of *Fam78a*, *Ppapdc3*, *Chrne*, *Kctd12*, and *Il12a* in exercise signaling pathways and its beneficial effect on health. Expression of *Mt1* and *Mt2* is reported to be up-regulated by EPS^34,35^. In this study, *Mt1* and *Mt2* expression was significantly up-regulated by EPS, and their expression gradually increased after acute maximal exercise. *Atf3*, which was up-regulated in the acute maximal and VWR exercise models, was up-regulated in rat soleus muscle after a single bout of aerobic exercise^36^, and in human after eccentric exercise^37,38^. *Mt1* and *Mt2* play a role in the defense against oxidative stress^34^, and *Atf3* is induced by stressors (e.g., hypoxia and endoplasmic reticulum stress)^39^. Our acute maximal exercise protocol forced mice to exercise until exhaustion, which triggers stress signaling pathways, including oxidative stress, thereby up-regulating the stress-response genes *Mt1*, *Mt2*, and *Atf3*. Genes with common expression patterns in EPS and in vivo exercise could be used as biomarkers of the effects of exercise on muscles.

We identified three transcriptional regulators associated with exercise, FOXM1, WNT5A, and AREG. FOXM1, a transcription factor that regulates cell proliferation and self-renewal, has been known to promote myogenesis in satellite cells^40^. The expression of WNT5A is up-regulated in mouse muscle during exercise^41^ and muscle regeneration^42^. Areg is reportedly up-regulated in human neutrophils 30 min after exercise^43^, and after acute resistance exercise in human skeletal muscle^31^. In injured mouse skeletal muscle, Areg is induced in regulatory T (Treg) cells and acts directly on muscle satellite cells to enhance muscle repair^44^. The expression of *Areg* was increased in remobilized soleus muscle at 10 days after hindlimb immobilization in young rats but not in old rats^45^. *Areg* expression is up-regulated in myotubes overexpressing PPARGC1A^46^, which is associated with muscle adaptation to endurance exercise^47^. Areg, a member of the EGF family that binds epidermal growth factor receptor (EGFR), is expressed in a variety of cell types, including muscle satellite cells and myoblasts^48,49^. EGF stimulates asymmetric satellite cell division by activating EGFR-Aurora kinase A (Aurka) signaling^50,51^. According to STRING analysis, target molecules of Areg analyzed by IPA (Supplementary Table 2) are associated with cell cycle. Notably, in this study, *Areg* expression was induced in both EPS myotubes and exercised mouse muscles, implicating myokine. Based on our findings, Areg is secreted from muscle after exercise and has the potential to enhance muscle repair by activating EGFR signaling and regulating the cell cycle. Further research is needed to determine underlying molecular mechanism. Other myokine candidates in Table 2 include neural EGFL like 2 (*Nell2*), which is up-regulated in exercised mice muscle^52^, and nidogen-1 (*Nid1*), which is up-regulated in exercised human muscle^53,54^, also need further research as a novel myokine.

The publicly available datasets used in this study focused on endurance exercise and can be classified into voluntary exercise (GSE70857, GSE31839, GSE31843), acute exercise (GSE79739, GSE79907, GSE97415), and chronic exercise (GSE126001, GSE104079, GSE75869). Although there were genes that tended to be up- or down-regulated only by a specific type of exercise, we identified common genes involved in various exercises and EPS, irrespective of exercise type. Additional research to elucidate exercise type similar to the in vitro exercise system at the transcriptional level would be informative.

EPS facilitates the discovery of intrinsic or autocrine factors of muscle without paracrine or endocrine effects on other organs. However, because exercise has a systemic effect, EPS cannot detect changes in gene expression driven by organ-to-muscle communication. In this study, several genes showed different expression patterns between EPS and in vivo exercise. This could be because EPS cannot represent the systemic effect of in vivo exercise. Studies involving organ-to-muscle communication by co-culturing muscle cells with other cell types^55^ and using EPS-conditioned medium^56–58^ have been conducted to address these differences. Although our findings demonstrated that EPS mimics in vivo exercise transcriptionally, it does not represent the metabolic aspect of in vivo exercise. Further research is needed to analyze the differences in metabolites between EPS and in vivo exercise. In conclusion, EPS mimics in vivo exercise, enabling us to identify putative exercise signature genes including Areg as a novel myokine. EPS could aid the discovery of novel myokines and would be useful in studies of responses to exercise.

## Methods

### Cell culture

C2C12 cells (ATCC, Manassas, VA, USA) were cultured in Dulbecco’s modified Eagle’s medium (DMEM; Gibco, Grand Island, NY, USA) with antibiotics and 10% fetal bovine serum (FBS; Gibco). C2C12 cells were plated into six-well plates for EPS. When the C2C12 cells reached approximately 80–90% confluence, medium was switched from 10% FBS medium to 2% FBS medium with antibiotics to induce differentiation. The medium was changed once every 2 days.

### Electrical pulse stimulation

Three days after differentiation, C2C12 myotubes in six-well plates were subjected to EPS at 37℃ using a C-PACE EP Culture Pacer (IonOptix, Dublin, Ireland). EPS was applied as a 1 ms pulse stimulus of 2 ms duration at 11.5 V and 1 Hz for 24 h, followed by 1 h of rest. EPS-conditioned medium with and without EPS were collected for further experiments.

### Immunofluorescence

C2C12 myoblasts were seeded on six-well plates with coverslips and differentiated into myotubes. EPS was applied for 24 h. After 1 h of rest, myotubes were rinsed in phosphate-buffered saline (PBS) and fixed in 4% paraformaldehyde for 15 min. After washing three times in PBS, myotubes were permeabilized in 0.1% Triton X-100 in PBS for 15 min. Myotubes were blocked with 0.2% bovine serum albumin (BSA) for 1 h and incubated with rhodamine phalloidin (Invitrogen, Carlsbad, CA, USA) overnight at 4 °C. Coverslips were mounted in DAPI mounting medium using Vectashield. Confocal fluorescence images of C2C12 myotubes were acquired with an Olympus FluoView FV1000. Intensity of F-actin were quantified using FV10-ASW software (v.4.2).

### RNA isolation

Total RNA was extracted from C2C12 myotubes using an easy-BLUE™ Total RNA Extraction Kit according to the manufacturer’s instructions (iNtRON Biotechnology, Seongnam, Korea). RNA quality was assessed using the Agilent 2100 Bioanalyzer (Agilent Technologies, Amstelveen, The Netherlands), and RNA quantification was performed using an ND-2000 Spectrophotometer (Thermo Fisher Scientific, Waltham, MA, USA).

### Library preparation and sequencing

Libraries were prepared from total RNA using the NEBNext Ultra II Directional RNA-Seq Kit (New England BioLabs, Ipswich, MA, USA). mRNA was isolated using the Poly(A) RNA Selection Kit (Lexogen, Vienna, Austria). mRNA was used for cDNA synthesis and shearing following the manufacturer’s instructions. Indexing was performed using Illumina indices 1–12. Enrichment was carried out by PCR. Subsequently, libraries were checked using the Agilent 2100 Bioanalyzer and DNA High-Sensitivity Kit to evaluate the mean fragment size. Quantification was performed using the Library Quantification Kit and StepOne™ Real-Time PCR System (Life Technologies, Carlsbad, CA, USA). High-throughput sequencing was performed on paired-end reads (2 × 100 bp) using the HiSeq 2500 platform (Illumina, San Diego, CA, USA.).

### Transcriptome profiling

Reference genome sequence data from *Mus musculus* were obtained from the NCBI Genome Database (assembly ID: GRCm38). Reference genome indexing and read mapping of tissue samples were performed using STAR software (ver. 2.5.4b). The CPM of each sample were determined by analyzing the RNA-seq data. Gene expression data were log2-transformed and quantile-normalized. To estimate the significance of differences in gene expression between groups, the edgeR package, which uses a negative binomial model, was employed to detect DEGs based on count data^59^. Expression differences were considered statistically significant if the *P*-value was < 0.001 and the fold difference in expression was ≥ 2. The RNA-seq dataset is available in the National Center for Biotechnology Information Gene Expression Omnibus database under accession number GSE176563.

### Upstream regulator analysis

To identify upstream regulators responsible for gene expression changes, upstream regulator analysis was performed using the Ingenuity Pathway Analysis tool. This analysis determined the number of known targets of each regulator and compared their direction of change to that expected based on previous reports. An overlap *P*-value and activation Z-score were estimated for each potential regulator. The overlap *P*‐value, estimated by Fisher’s exact test, indicates whether there is statistically significant overlap between the genes in a dataset and genes regulated by a regulator. The activation Z-score is used to infer the likely activation states of regulator candidates by comparison with a model that assigns a “random regulation direction”. Positive and negative activation Z-scores indicate that a potential upstream regulator is activated and inhibited, respectively.

### Development of the prediction model

To validate the predictive value of the in vitro data, a prediction model was generated using a DBN^26^ algorithm. Briefly, the model incorporated 774 DEGs between the EPS and unstimulated control groups, determined by tests performed using the EdgeR package. To construct a fully connected neural network, four hidden layers, in which 800, 500, 300, and 100 nodes were allocated, were used. When training the model, a Tanh function was used as the activation function, and the training procedure was repeated over 5,000 epochs. To prevent overfitting or non-convergence of the classifier, the prediction model was pre-trained by an auto-encoder^26^ algorithm. The prediction model was undertaken using the H2O deep-learning platform (ver. 3.32.0.2; https://www.h2o.ai). The code snippet (in R language) for the current investigation is as follows:

- *# step of auto-encoding*
- *m1 <- h2o.deeplearning(1:785, training_frame  =  data_train, hidden  =  c(800,500,300,100), autoencoder  =  T, activation  =  "Tanh", epochs  =  5000)*
- *# step of training*
- *m2 <- h2o.deeplearning(x, y, training_frame  =  data_train, hidden  =  c(800,500,300,100), pretrained_autoencoder  =  m1@model_id, activation  =  "Tanh", epochs  =  5000)*

### Publicly available in vivo exercise datasets

We searched mouse exercise GEO datasets GSE70857, GSE31839, GSE31843, GSE79739, GSE79907, GSE97415, GSE126001, GSE104079, and GSE75869 for various muscle types and exercise protocols. The datasets are outlined in Table 1.

### Quantitative RT-PCR

RNA preparation and cDNA synthesis were performed according to standard protocols. Quantitative RT-PCR analysis was performed using the StepOnePlus™ Kit (Applied Biosystems, Foster City, CA, USA). The 36B4 mRNA level was used for normalization. The primers are listed in Supplementary Table 4.

### Animal experiments

Three-month-old female and male C57BL/6 mice were purchased from Damul Science (Daejeon, South Korea). Mice were given ad libitum access to food and water, and were maintained on a 12 h light: 12 h dark cycle. The mice were familiarized with running on a treadmill for 10 min on the day before exercise. For acute maximal exercise, female mice were randomized into three groups; sedentary group (n = 16) that did not perform exercise and 0 h resting (n = 16) and 1 h resting (n = 16) according to the resting time after exercise. The acute maximal exercise was described previously^60^. Briefly, the first 5 min of the treadmill exercise was performed at a rate of 13 m/min, which then increased by 1 m/min to 14 m/min. The mice ran at 18 m/min for the next 30 min. After 40 min, the speed was increased to a maximum of 23 m/min in 1 m/min increments. Exercise continued until exhaustion (up to 2 h). The acute maximal exercise with longer resting time was performed independently with the same exercise protocol. For short-term chronic exercise, male mice were randomized into short-term chronic (n = 8) and sed (n = 8) groups. Mice performed 5 days of exercise on a treadmill for 60 min/day. The short-term chronic exercise started at 10 m/min and increased up to 20 m/min at 10-min intervals. For long-term chronic exercise, male mice were randomized into long-term chronic (n = 8) and sed (n = 8) groups. Mice performed 4 weeks of chronic exercise training on a treadmill, running for 5 days/week for 30 min/day at 12 m/min. For the VWR exercise, male mice were randomized into VWR (n = 8) and sed-VWR (n = 8) groups. The mice were housed individually in cages. The 8 mice in the VWR group were provided with an in-cage running wheel. Running wheel activity was monitored daily for 4 weeks. At the end of each experiment, the gastrocnemius (GA) and tibialis anterior (TA) muscles were removed for qRT-PCR analysis. The mice were maintained in accordance with the ARRIVE guidelines and animal care guidelines of Animal Care and Use Committee of KRIBB. All animal experiments were approved by the Animal Care and Use Committee of KRIBB.

### Enzyme-linked immunosorbent assay

EPS-conditioned medium was subjected to enzyme-linked immunosorbent assay (ELISA), to evaluate AREG secretion using the DuoSet ELISA Development kit against mouse AREG (R&D Systems, Minneapolis, MN, USA) following the manufacturer’s protocol.

### Statistical analysis

Data are presented as means ± SEM unless indicated otherwise. The two-tailed unpaired Student’s *t*-test was performed using GraphPad Prism software (GraphPad Software Inc., La Jolla, CA, USA). *P*-values < 0.05 were considered indicative of statistical significance.

### Ethics declarations

All methods were performed in accordance with the relevant guidelines and regulations.

## Supplementary Information

Supplementary Figures.

Supplementary Table 1.

Supplementary Table 2.

Supplementary Table 3.

Supplementary Table 4.

## Data Availability

The datasets generated and/or analysed during the current study are available in the [https://www.ncbi.nlm.nih.gov/geo/ GSE176563] repository.

## References

[1] Karstoft K, Pedersen BK (2016). Exercise and type 2 diabetes: Focus on metabolism and inflammation. Immunol. Cell Biol..

[2] Fiuza-Luces C (2018). Exercise benefits in cardiovascular disease: Beyond attenuation of traditional risk factors. Nat. Rev. Cardiol..

[3] Panza GA (2018). Can exercise improve cognitive symptoms of Alzheimer's disease?. J. Am. Geriatr. Soc..

[4] Kurgan N (2017). Inhibition of human lung cancer cell proliferation and survival by post-exercise serum is associated with the inhibition of Akt, mTOR, p70 S6K, and Erk1/2. Cancers.

[5] Dethlefsen C (2017). Exercise-induced catecholamines activate the hippo tumor suppressor pathway to reduce risks of breast cancer development. Can. Res..

[6] Pedersen L (2016). Voluntary running suppresses tumor growth through epinephrine-and IL-6-dependent NK cell mobilization and redistribution. Cell Metab..

[7] Hojman P, Gehl J, Christensen JF, Pedersen BK (2018). Molecular mechanisms linking exercise to cancer prevention and treatment. Cell Metab..

[8] Idorn M, Hojman P (2016). Exercise-dependent regulation of NK cells in cancer protection. Trends Mol. Med..

[9] Severinsen MCK, Pedersen BK (2020). Muscle–organ crosstalk: The emerging roles of myokines. Endocr. Rev..

[10] Castillo-Armengol J, Fajas L, Lopez-Mejia IC (2019). Inter-organ communication: A gatekeeper for metabolic health. EMBO Rep..

[11] Boström P (2012). A PGC1-α-dependent myokine that drives brown-fat-like development of white fat and thermogenesis. Nature.

[12] Jedrychowski MP (2015). Detection and quantitation of circulating human irisin by tandem mass spectrometry. Cell Metab..

[13] Thelen MH, Simonides WS, Hardeveld CV (1997). Electrical stimulation of C2C12 myotubes induces contractions and represses thyroid-hormone-dependent transcription of the fast-type sarcoplasmic-reticulum Ca^2+^-ATPase gene. Biochem. J..

[14] Lautaoja JH (2021). Higher glucose availability augments the metabolic responses of the C2C12 myotubes to exercise-like electrical pulse stimulation. Am. J. Physiol. Endocrinol. Metab..

[15] Nikolic N (2013). Correction: Electrical pulse stimulation of cultured human skeletal muscle cells as an in vitro model of exercise. PLoS ONE.

[16] Burch N (2010). Electric pulse stimulation of cultured murine muscle cells reproduces gene expression changes of trained mouse muscle. PLoS ONE.

[17] Nedachi T, Fujita H, Kanzaki M (2008). Contractile C2C12 myotube model for studying exercise-inducible responses in skeletal muscle. Am. J. Physiol.-Endocrinol. Metab..

[18] Nedachi T, Hatakeyama H, Kono T, Sato M, Kanzaki M (2009). Characterization of contraction-inducible CXC chemokines and their roles in C2C12 myocytes. Am. J. Physiol.-Endocrinol. Metab..

[19] Lee HJ (2019). ATP synthase inhibitory factor 1 (IF1), a novel myokine, regulates glucose metabolism by AMPK and Akt dual pathways. FASEB J..

[20] Raschke S, Eckardt K, Bjørklund Holven K, Jensen J, Eckel J (2013). Identification and validation of novel contraction-regulated myokines released from primary human skeletal muscle cells. PLoS ONE.

[21] Broholm C (2011). LIF is a contraction-induced myokine stimulating human myocyte proliferation. J. Appl. Physiol..

[22] Gorgens SW (2016). The exercise-regulated myokine chitinase-3-like protein 1 stimulates human myocyte proliferation. Acta Physiol. (Oxf.).

[23] Gonzalez-Franquesa A (2021). Discovery of thymosin beta4 as a human exerkine and growth factor. Am. J. Physiol. Cell Physiol..

[24] Takahashi T (2022). RSPO3 is a novel contraction-inducible factor identified in an “in vitro exercise model” using primary human myotubes. Sci. Rep..

[25] Fujita H, Nedachi T, Kanzaki M (2007). Accelerated de novo sarcomere assembly by electric pulse stimulation in C2C12 myotubes. Exp. Cell Res..

[26] Hinton GE, Osindero S, Teh Y-W (2006). A fast learning algorithm for deep belief nets. Neural Comput..

[27] Vianney J-M, Miller DA, Spitsbergen JM (2014). Effects of acetylcholine and electrical stimulation on glial cell line-derived neurotrophic factor production in skeletal muscle cells. Brain Res..

[28] Aas V, Torblå S, Andersen MH, Jensen J, Rustan AC (2002). Electrical stimulation improves insulin responses in a human skeletal muscle cell model of hyperglycemia. Ann. N. Y. Acad. Sci..

[29] Son YH (2019). Comparative molecular analysis of endurance exercise in vivo with electrically stimulated in vitro myotube contraction. J. Appl. Physiol..

[30] Nikolić N (2012). Electrical pulse stimulation of cultured human skeletal muscle cells as an in vitro model of exercise. PLoS ONE.

[31] Pillon NJ (2020). Transcriptomic profiling of skeletal muscle adaptations to exercise and inactivity. Nat. Commun..

[32] Ramirez-Martinez A (2021). The nuclear envelope protein Net39 is essential for muscle nuclear integrity and chromatin organization. Nat. Commun..

[33] Meinke P (2020). A multistage sequencing strategy pinpoints novel candidate alleles for Emery-Dreifuss muscular dystrophy and supports gene misregulation as its pathomechanism. EBioMedicine.

[34] Ruttkay-Nedecky B (2013). The role of metallothionein in oxidative stress. Int. J. Mol. Sci..

[35] Evers-van Gogh IJ (2015). Electric pulse stimulation of myotubes as an in vitro exercise model: Cell-mediated and non-cell-mediated effects. Sci. Rep..

[36] McKenzie MJ, Goldfarb AH (2007). Aerobic exercise bout effects on gene transcription in the rat soleus. Med. Sci. Sports Exerc..

[37] Chen YW, Hubal MJ, Hoffman EP, Thompson PD, Clarkson PM (2003). Molecular responses of human muscle to eccentric exercise. J. Appl. Physiol..

[38] MacNeil LG, Melov S, Hubbard AE, Baker SK, Tarnopolsky MA (2010). Eccentric exercise activates novel transcriptional regulation of hypertrophic signaling pathways not affected by hormone changes. PLoS ONE.

[39] Hai T, Wolford CC, Chang YS (2010). ATF3, a hub of the cellular adaptive-response network, in the pathogenesis of diseases: Is modulation of inflammation a unifying component?. Gene Expr.

[40] Chen Z (2020). A Cdh1–FoxM1–Apc axis controls muscle development and regeneration. Cell Death Dis..

[41] Fujimaki S, Hidaka R, Asashima M, Takemasa T, Kuwabara T (2014). Wnt protein-mediated satellite cell conversion in adult and aged mice following voluntary wheel running. J. Biol. Chem..

[42] Polesskaya A, Seale P, Rudnicki MA (2003). Wnt signaling induces the myogenic specification of resident CD45+ adult stem cells during muscle regeneration. Cell.

[43] Radom-Aizik S, Zaldivar F, Leu S-Y, Galassetti P, Cooper DM (2008). Effects of 30 min of aerobic exercise on gene expression in human neutrophils. J. Appl. Physiol..

[44] Burzyn D (2013). A special population of regulatory T cells potentiates muscle repair. Cell.

[45] Pattison JS, Folk LC, Madsen RW, Booth FW (2003). Selected contribution: Identification of differentially expressed genes between young and old rat soleus muscle during recovery from immobilization-induced atrophy. J. Appl. Physiol..

[46] Re Cecconi AD (2019). Musclin, a myokine induced by aerobic exercise, retards muscle atrophy during cancer cachexia in mice. Cancers (Basel).

[47] Arany Z (2008). PGC-1 coactivators and skeletal muscle adaptations in health and disease. Curr. Opin. Genet. Dev..

[48] Lazure F (2020). Myf6/MRF4 is a myogenic niche regulator required for the maintenance of the muscle stem cell pool. EMBO Rep..

[49] De Micheli AJ, Spector JA, Elemento O, Cosgrove BD (2020). A reference single-cell transcriptomic atlas of human skeletal muscle tissue reveals bifurcated muscle stem cell populations. Skelet. Muscle.

[50] Wang YX (2019). EGFR-Aurka signaling rescues polarity and regeneration defects in dystrophin-deficient muscle stem cells by increasing asymmetric divisions. Cell Stem Cell.

[51] Feige P, Tsai EC, Rudnicki MA (2021). Analysis of human satellite cell dynamics on cultured adult skeletal muscle myofibers. Skelet. Muscle.

[52] Endo Y (2021). Exercise-induced gene expression changes in skeletal muscle of old mice. Genomics.

[53] Williams K (2021). Epigenetic rewiring of skeletal muscle enhancers after exercise training supports a role in whole-body function and human health. Mol. Metab..

[54] Hjorth M (2015). The effect of acute and long-term physical activity on extracellular matrix and serglycin in human skeletal muscle. Physiol. Rep..

[55] Nintou E (2021). Effects of in vitro muscle contraction on thermogenic protein levels in co-cultured adipocytes. Life (Basel).

[56] Laurens C (2020). Growth and differentiation factor 15 is secreted by skeletal muscle during exercise and promotes lipolysis in humans. JCI Insight.

[57] Barlow J, Solomon TPJ (2019). Conditioned media from contracting skeletal muscle potentiates insulin secretion and enhances mitochondrial energy metabolism of pancreatic beta-cells. Metabolism.

[58] Schwappacher R (2020). Physical activity and advanced cancer: Evidence of exercise-sensitive genes regulating prostate cancer cell proliferation and apoptosis. J. Physiol..

[59] Robinson MD, McCarthy DJ, Smyth GK (2010). edgeR: A bioconductor package for differential expression analysis of digital gene expression data. Bioinformatics.

[60] Reynolds JC (2021). MOTS-c is an exercise-induced mitochondrial-encoded regulator of age-dependent physical decline and muscle homeostasis. Nat. Commun..

