# Supplementary material for: Genome-wide analysis of a cellular exercise model based on electrical pulse stimulation
Source: Sci Rep. 2022 Dec 8;12:21251. doi: 10.1038/s41598-022-25758-2 (PMC9731977; doi:10.1038/s41598-022-25758-2)
Supplement: Supplementary file 1 — Supplementary Figures. [file 41598_2022_25758_MOESM1_ESM.pdf]

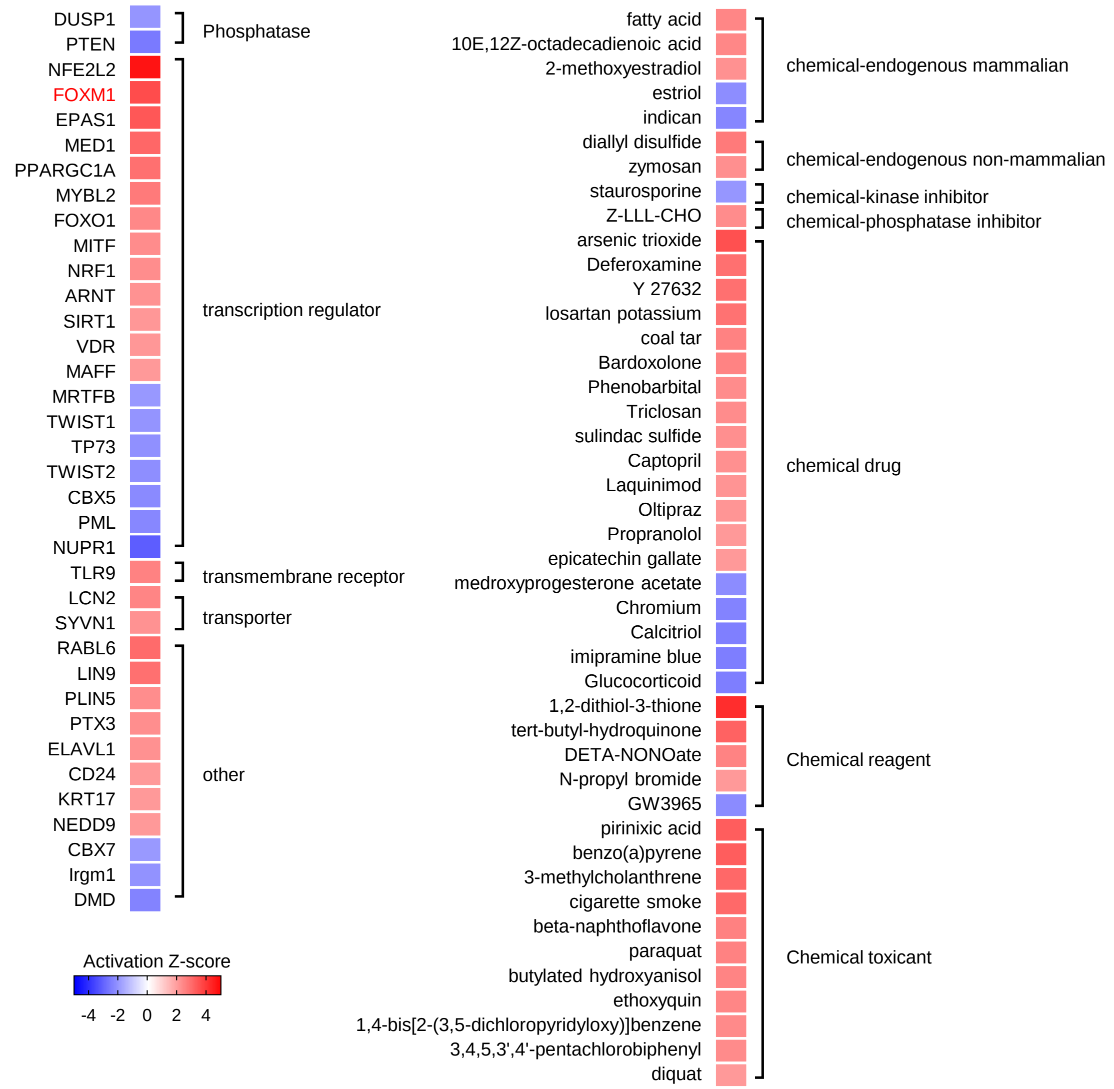

Supplementary Fig. 1. Predicted upstream regulators in EPS. Activation Z-scores ≥ 2 or ≤ -2 and $P < 0.05$.

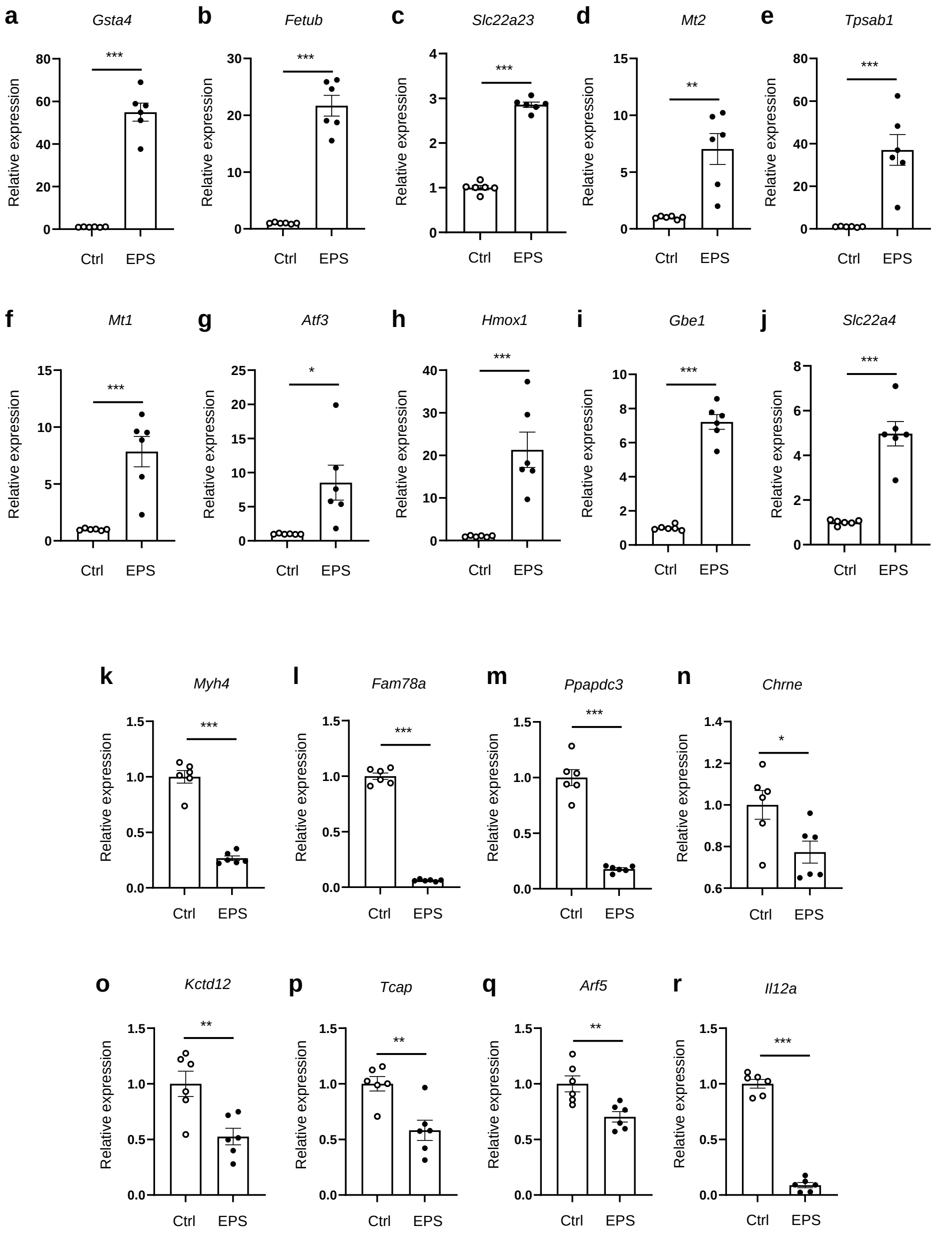

Supplementary Fig. 2. Validation of the top 10 up- and down-regulated genes in EPS samples. (**a-j**) Validation of the top 10 up-regulated genes. (**k-r**) Validation of the top 10 down-regulated genes except low-abundance genes.

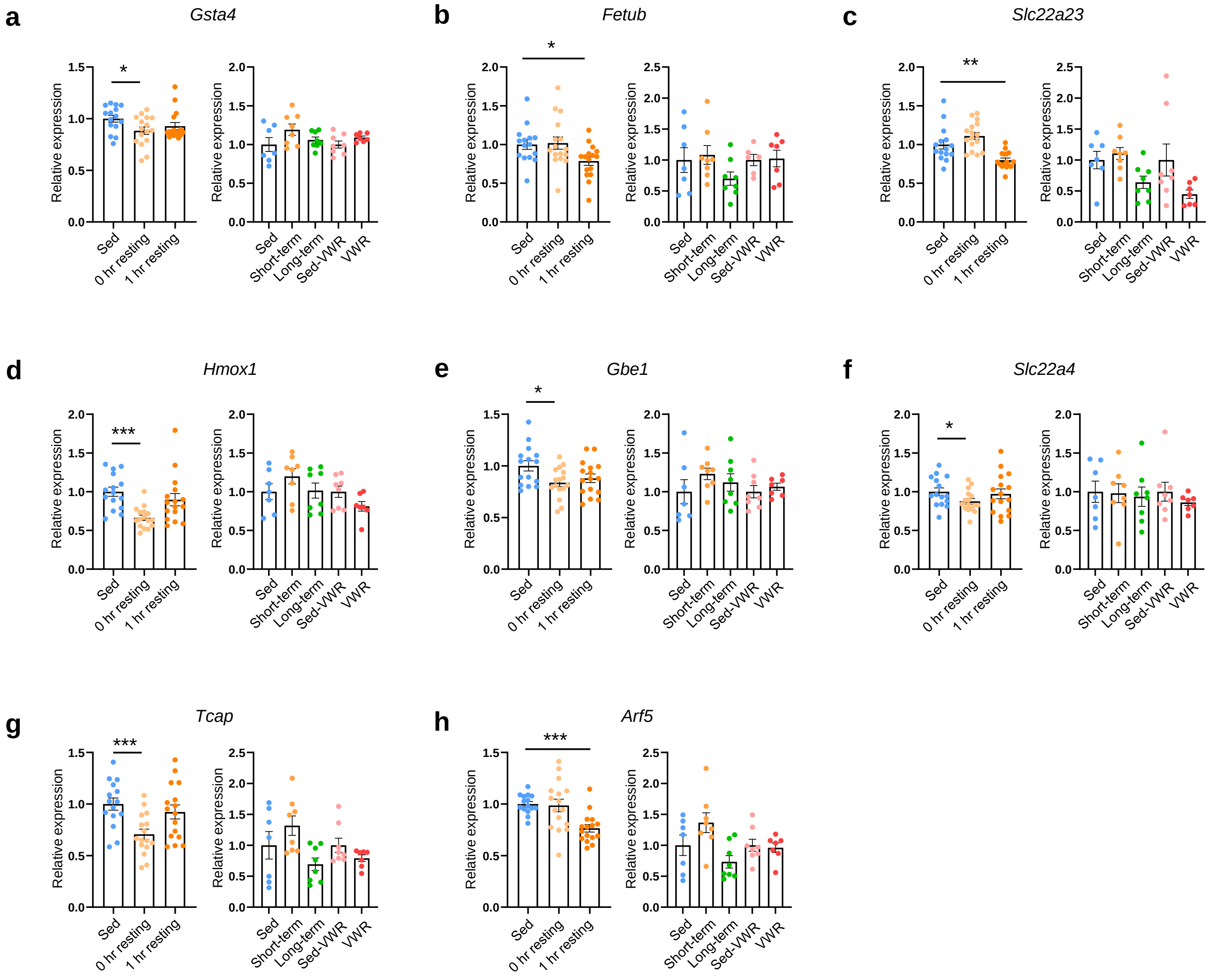

Supplementary Fig. 3. Expression of DEGs in exercised muscles. *Gsta4*, *Fetub*, *Slc22a23*, *Hmox1*, *Gbe1*, *Slc22a4*, *Tcap* and *Arf5* expression following the indicated types of exercise. For acute maximal exercise, sed (n = 15), 0 h rest (n = 16), and 1 h rest (n = 16) TA muscles were analyzed. For chronic exercise, sed (n = 7), short-term (n = 8), and long-term (n = 8) GA muscles were analyzed. For VWR exercise, sed-VWR (n = 8) and VWR (n = 7) GA muscles were analyzed. Means ± SEM; *P < 0.05, **P < 0.01, ***P < 0.001. *Tpsab1* was not detected because of its low abundance in muscle tissues.
