# Supplementary material for: Genome-wide analysis of a cellular exercise model based on electrical pulse stimulation
Source: Sci Rep. 2022 Dec 8;12:21251. doi: 10.1038/s41598-022-25758-2 (PMC9731977; doi:10.1038/s41598-022-25758-2)
Supplement: Supplementary file 5 — Supplementary Table 4. [file 41598_2022_25758_MOESM5_ESM.docx]

Supplementary Table 4. Primer sequences for RT-qPCR.

| Gene | Sequence (5’- to - 3’) | Reverse (5’- to - 3’) |
| --- | --- | --- |
| *Gsta4* | GATGATTGCCGTGGCTCCATTTA | CTGGTTGCCAACGAGAAAAGCC |
| *Fetub* | ACGGTCCAAAGTTCTCTGAGGC | CTGGGTAACAGCAGGGTTCTCA |
| *Slc22a23* | GTCTGGTAGTCAAGTTCCTCGG | CTGAGTCTGGATGCTGGCTGTA |
| *Mt2* | CGTGGGCTGTGCGAAGTGCTC | AAAGGCTAGGCTTCTACATGGTC |
| *Tpsab1* | CCTCGGGCTGTTGGAATGAA | AGTTGGAGGTGAGGGCTAGT |
| *Mt1* | CAAGAACTGCAAGTGCACCTC | CGCCTTTGCAGACACAGC |
| *Atf3* | GAAGATGAGAGGAAAAGGAGGCG | GCTCAGCATTCACACTCTCCAG |
| *Hmox1* | CACTCTGGAGATGACACCTGAG | GTGTTCCTCTGTCAGCATCACC |
| *Gbe1* | CTCTGTTCTCCAACTTCCCAGG | CAAGGTAGCGTCGATTGGTGAG |
| *Slc22a4* | GCCGCAAAGATGAACAGCATCG | CGGTTATGGTGGCAATGTTCCG |
| *Myh4* | AGAGCCAAGAGGAAACTGGAGG | CTCGTCCTCAATCTTGCTCTGC |
| *Fam78a* | AGCAGGGCATGTCTAGCTGG | CACGTGGTGAAGCTCTGGTC |
| *Arx* | CAGTTACCAGCTGGAGGAACT | CCGACGGTTCTGGAACCACA |
| *Ppapdc3* | ATCCTCTGCCTGGTGAGAAGCA | TTCTGGACTCCAGCCACTGTCA |
| *Chrne* | AGACCTGAGGACACTGTCACCA | TCGTCCTTGCTGTAGTTGAGCC |
| *Kctd12* | AGTCAAGTGATGTCAAACCGAC | GGATCAGTGAGCAAGGATTCTTT |
| *Tcap* | TTCTGGGCTGAGTGGAAAGACC | GCTGTACCACCGCCTGACACT |
| *Arf5* | GCCTCATCTTTGTGGTAGACAGC | GTCCTGCTTGTTGGCAAACACC |
| *Il12a* | ACGAGAGTTGCCTGGCTACTAG | CCTCATAGATGCTACCAAGGCAC |
| *Rtn4r* | Mm00452228_m1 | |
| *Areg* | Mm00437583_m1 | |
| *36B4* | AGATTCGGGATATGCTGTTGG | AAAGCCTGGAAGAAGGAGGTC |
